# Association of polymorphisms in *survivin *gene with the risk of hepatocellular carcinoma in Chinese han population: a case control study

**DOI:** 10.1186/1471-2350-13-1

**Published:** 2012-01-03

**Authors:** Yuhua Li, Jiaofeng Wang, Feng Jiang, Wenyao Lin, Wei Meng

**Affiliations:** 1 Department of Epidemiology, School of Public Health, Fudan University; Key Laboratory of Public Health Security, Ministry of Education, Shanghai, 200032, China; 2 Changning Center for Disease Control and Prevention, Shanghai, 200051, China; 3 Haimen Center for Disease Control and Prevention, Jiangsu, 226100, China

## Abstract

**Background:**

Survivin, one of the strongest apoptosis inhibitors, plays a critical role in the development and progression of hepatocellular carcinoma (HCC). By comparison, relatively little is known about the effect of *survivin *gene polymorphisms on HCC susceptibility. Our study aimed to investigate the association of *survivin *gene polymorphisms with the risk of HCC in Chinese han population.

**Methods:**

A case-control study was conducted in Chinese han population consisting of 178 HCC cases and 196 cancer-free controls. Information on demographic data and related risk factors was collected for all subjects. Polymorphisms of the *survivin *gene, including three loci of rs8073069, rs9904341 and rs1042489, were selected and genotyped by a polymerase chain reaction- restriction fragment length polymorphism (PCR-RFLP) technique. Association analysis of genotypes/alleles and haplotypes from these loci with the risk of HCC was conducted under different genetic models.

**Results:**

Using univariate analysis of rs8073069, rs9904341 and rs1042489 under different genetic models, no statistically significant difference was found in genotype or allele distribution of HCC cases relative to the controls (*P *> 0.05). Linkage disequilibrium (LD) analysis showed that these loci were in LD. Multivariate logistic regression indicated that with no G-C-T haplotype as reference, the haplotype of G-C-T from these loci was associated with a lower risk for HCC under the recessive model (*OR = *0.46, 95% confidence interval (*CI*): 0.24~0.90, *P *= 0.023). Both HBsAg+ and the medical history of viral hepatitis type B were risk factors for HCC. However, no statistically significant haplotype-environment interaction existed.

**Conclusions:**

No association between rs8073069, rs9904341 or rs1042489 in *survivin *gene and the risk of HCC is found in Chinese han population, but rs8073069G-rs9904341C- rs1042489T is perhaps a protective haplotype for HCC.

## Background

Hepatocellular carcinoma (HCC) is the sixth most common cancer in the world and the third most common cause of cancer-related death, which represents a major and constantly rising health burden throughout the world. In addition, HCC shows great geographical variation, with a very high incidence in China, where approximately 55% of annual new cases worldwide emerge [1,2]. Nowadays, the exact mechanism of hepatocarcinogenesis is still incompletely understood. However, a number of relevant molecules alter in some biological signals in the preneoplastic hepatocytes, especially the overactivation of anti-apoptotic signals, which disrupts the balance between survival and apoptotic signals [3]. It's widely accepted that apoptosis plays a key role in cell or tissue homeostasis [4,5], dysregulation of apoptosis may induce the accumulation of virtually immortal cells and can ultimately lead to many human disorders, including cancer [6].

Survivin is a novel member of the inhibitor of apoptosis family of proteins (IAPs), containing a single baculovirus IAP repeat (BIR) domain [7]. It is involved in cell cycle regulation, inhibition of the apoptosis pathways and microtubule stability [8], playing a critical role in the initiation and progression of tumorigeness. Survivin blocks both death receptor and mitochondrial apoptosis pathways, by directly inhibiting caspase-3 and caspase-7 as well as by interfering with caspase-9 activity/processing [9]. Furthermore, survivin inhibits apoptosis initiated by various apoptotic stimuli such as IL-3, Fas, Bax, TNF-α, anticancer drugs, and X-irradiation [10,11]. In addition, it's reported that survivin was associated with angiogenesis [12], which is critical in carcinogenesis. Survivin is ubiquitous in embryonic or fetal tissues while undetectable in most terminally differentiated normal adult tissues [7]. By contrast, it is overexpressed in most human cancers including HCC [13-17]. Moreover, increased survivin expression in human malignancies is considered to be an important marker for aggressive and chemoresistant disease, thus signaling poor prognosis [8,18-21].

Survivin is expressed in a cell cycle-regulated manner, with a peak in the G2/M phase of the cell cycle, while it rapidly declines in the G1 phase [22]. This is largely transcriptionally controlled and involves cell cycle-dependent elements (CDEs) and cell cycle homology regions (CHRs) located in *survivin *gene promoter [23]. Several single nucleotide polymorphisms (SNPs) have been identified in *survivin *gene, such as -31G/C, -625G/C and -644C/T. -31G/C polymorphism is a common mutation in cancer cell lines leading to overexpression of survivin and the aberrant cell cycle-dependent transcription, mediated via functional disruption of binding at the CDE/CHR repressor motifs [24]. Several population-based studies indicated *survivin *gene polymorphisms were associated with human cancers [25-27]. However, to date, there was only one report on the relationship between *survivin *gene polymorphisms and the risk of HCC [28]. No similar study has been conducted yet in Chinese population.

Based on the key role of survivin in carcinogenesis and the association of *survivin *gene polymorphisms with its expression and other cancers, we hypothesized that polymorphisms in *survivin *gene might modulate susceptibility to HCC. To test this hypothesis, we investigated the association between *survivin *gene polymorphisms and the risk of HCC in Chinese han population.

## Methods

### Study Population and Samples Collection

This case-control study consisted of 178 HCC patients and 196 cancer-free controls. All subjects were unrelated han nationality living in Haimen city, Jiangsu province. HCC patients were diagnosed by doctors according to the standards established by Chinese Society of Liver Cancer (CSLC) [29]. Controls were matched with the patients in terms of age and sex, excluding those with medical history of surgery or chronic disease. Each subject was personally face-to-face interviewed by trained interviewers for information on demographic data as well as related risk factors such as tobacco smoking, alcohol drinking, medical history of viral hepatitis type B, et al. In addition, approximately 5 ml venous blood was drawn from each subject and preserved at -80°C. This research protocol was approved by the Ethics Committee of Fudan University, with the number IRB# 08-08-0142.

### DNA Extraction and Genotyping

**Figure 1:**
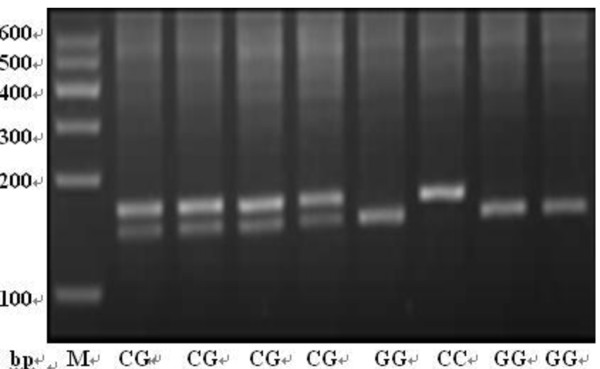
**The genotypes of rs8073069 by PCR-RFLP**.

**Figure 2:**
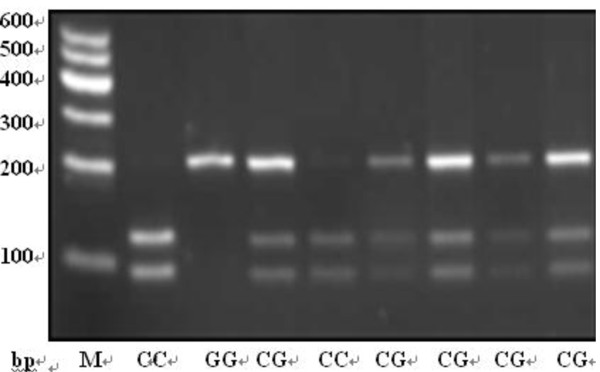
**The genotypes of rs9904341 by PCR-RFLP**.

**Figure 3:**
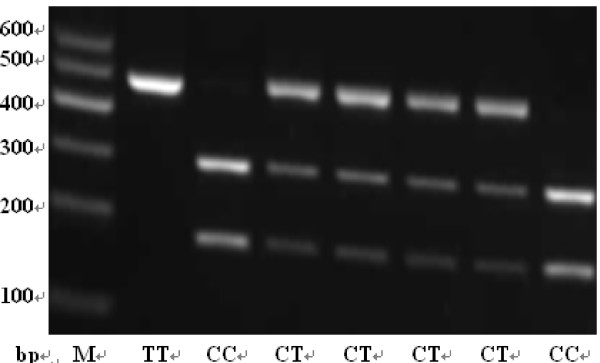
**The genotypes of rs1042489 by PCR-RFLP**.

The genomic DNA was extracted from each blood sample using RelaxGenne Blod DNA system (TIANGEN, Beijing). Each polymorphism of rs8073069, rs9904341 and rs1042489 was identified via the polymerase chain reaction-restriction fragment length polymorphism (PCR-RFLP) technique. The primers for PCR, the restriction enzymes, and the fragments length after digestion are shown in Table 1. Amplification was carried out on a GeneAmp PCR System PTC-200 (MJ, American), PCR mixture contained 50 ng of DNA, 9.5 μl ddH_2_O, 12.5 μl 2 × Taq PCR Master Mix (Laifeng, Shanghai), 1 μl 10 μmol/l each primer. The annealing temperature was 60.2°C, 64.4°C and 64.4°C for rs8073069, rs9904341 and rs1042489 respectively. PCR products were digested by the corresponding restriction enzymes (ChinaGen, Shenzhen) and then separated by 2.0~3.0% agarose gel electrophoresis with ethidium bromide (EB). The genotypes are shown in Figure 1, 2 and 3. For double-checking and quality control, 5% of the samples were randomly selected to perform the repeated assays, and the results were 100% concordant.

**Table 1:** PCR-RFLP-based assay of *survivin *SNPs

SNP	Position	Sequence of primer	Enzyme	Interpretation(bp)
rs8073069	-625	5'- GTYCATTTGTCCTTCATGCGC-3' 5'- GGCAGAGGGTGCAGTGAGC-3'	Bstu I	CC:164 CG:164,145 GG:145
rs9904341	-31	5'-GAGGACTACAACTCC CGGCAC-3' 5'- GTAGAGATGCGGTGGTCCTTG-3'	Msp I	GG:212,16 CC:120,92,16 CG:212,120,92,16
rs1042489	3'UTR	5'-GCTTACCAGGTGAGAAGTGAGG-3' 5'-GTATCTGCCAGACGCTTCCTATC-3'	Msp I	TT:476 TC:476,297,179 CC:297,179

### Statistical Analysis

Genotypes were tested for Hardy-Weinberg equilibrium by an online soft http://ihg.gsf.de/cgi-bin/hw/hwa1.pl. Differences in the distribution of genotypes or alleles under different genetic models (including dominant, recessive, additive and multiple models) within groups were estimated by univariate statistical analyses (chi-square test) on SPSS 16.0. Pair-wise linkage disequilibrium (LD) between SNPs was calculated by Haploview 4.1. In addition, haplotypes analysis was carried out by multivariate logistic regression analysis on Hapstat 3.0. The odds ratios (*OR*s) and 95% confidence intervals (*CI*s) were presented for association analysis. For all hypothesis tests, a two-tailed significance level of *P *< 0.05 was considered statistically significant.

## Results

### General Characteristics of the Subjects

A total of 374 subjects were recruited in our study, including 178 HCC cases and 196 controls. Their general characteristics are summarized in Table 2. There was no significant difference in age or gender between cases and controls (*P = *0.343 and 0.703 respectively). Besides, all subjects were local ethnic han population in Haimen, which indicated that both groups were comparable in demographic characteristics. With regard to the risk factors, Table 2 shows that the distributions of drinking, HBsAg+ and the medical history of viral hepatitis type B were significantly different within groups. Additionally, compared with the control subjects, the cases had a higher rate of smoke-quitting, but no difference was found between the proportion of subjects who never smoked and current smokers.

**Table 2:** General characteristics of the cases and controls

Factors		Case	Control	*P*	*OR*(95%*CI*)
Age(mean ± SD)		53.31 ± 8.41	52.48 ± 8.47	0.343	-
Sex n(%)	Male	136(76.4)	153(78.1)	-	-
	Female	42(23.6)	43(21.9)	0.703	-
Smoking n(%)	Never	71(39.9)	124(63.3)	-	1
	Current	47(26.4)	67(34.2)	0.400	1.23(0.76~1.97)
	Quitting	60(33.7)	5(2.6)	< 0.001	-
Drinking n(%)	No	101(56.7)	135(68.9)	-	1
	Yes	77(43.3)	61(31.1)	0.015	1.69(1.11~2.58)
HBsAg+ n(%)	No	48(27.0)	184(93.9)	-	1
	Yes	130(73.0)	12(6.1)	< 0.001	41.53(21.22~81.26)
H^a ^n(%)	No	105(59.0)	195(99.5)	-	1
	Yes	73(41.0)	1(0.5)	< 0.001	135.57(18.58~989.35)

^a ^The medical history of viral hepatitis type B

### *Survivin *Gene Polymorphisms of the Subjects

The genotype and allele frequencies of rs8073069, rs9904341, rs1042489 in both HCC cases and controls are presented in Table 3, 4 and 5. The genotype distributions of the three polymorphisms didn't significantly deviate from that expected for a Hardy-Weinberg equilibrium (for cases, *χ*^2 ^= 2.239, 2.820 and 2.675 respectively; for controls, *χ*^2 ^= 0.143, 0.078 and 1.451 respectively; all *P *values were higher than 0.05), illustrating that our subjects presented the source population well. We compared the genotype or allele frequencies of every polymorphism within groups under the dominant, recessive, additive and multiple genetic models respectively. As shown in Table 3, 4 and 5, no significant differences were detected in the distributions of genotypes or alleles between case and control groups.

**Table 3:** Frequency of rs8073069 genotypes/alleles under different genetic models

Genetic model	Genotypes/Alleles	Case n(%)	Control n(%)	*χ*^2^(*P*)	*OR*(95%*CI*)
Dominant	CC	16(9.0)	12(6.1)	-	1
	CG/GG	162(91.0)	184(93.9)	1.11(0.293)	0.66(0.30~1.44)
Recessive	CC/CG	77(43.3)	89(45.4)	-	1
	GG	101(56.7)	107(54.6)	0.18(0.676)	1.09(0.73~1.64)
Additive	CC	16(9.0)	12(6.1)	-	1
	CG	61(34.3)	77(39.3)	-	0.59(0.26~1.35)
	GG	101(56.7)	107(54.6)	1.74(0.420)	0.71(0.32~1.57)
Multiple	C	93(26.1)	101(25.8)	-	1
	G	263(73.9)	291(74.2)	0.01(0.911)	0.98(0.71~1.36)

**Table 4:** Frequency of rs9904341 genotypes/alleles under different genetic models

Genetic Model	Genotypes/Alleles	Case n(%)	Control n(%)	*χ*^2^(*P*)	*OR*(95%*CI*)
Dominant	CC	35(19.7)	52(26.5)	-	1
	CG/GG	143(80.3)	144(73.5)	2.47(0.116)	1.48(0.91~2.40)
Recessive	CC/CG	135(75.8)	148(75.5)	-	1
	GG	43(24.2)	48(24.5)	0.01(0.940)	0.98(0.61~1.58)
Additive	CC	35(19.7)	52(26.5)	-	1
	CG	100(56.2)	96(49.0)	-	1.55(0.93~2.58)
	GG	43(24.2)	48(24.5)	2.82(0.244)	1.33(0.74~2.41)
Multiple	C	170(47.8)	200(51.0)	-	1
	G	186(52.2)	192(49.0)	0.80(0.372)	1.14(0.86~1.52)

**Table 5:** Frequency of rs1042489 genotypes/alleles under different genetic models

Genetic Model	Genotypes/Alleles	Case n(%)	Control n(%)	*χ*^2^(*P*)	*OR*(95%*CI*)
Dominant	CC	31(17.4)	27(13.8)	-	1
	CT/TT	147(82.6)	169(86.2)	0.94(0.331)	0.76(0.43~1.33)
Recessive	CC/CT	130(73.0)	129(65.8)	-	1
	TT	48(27.0)	67(34.2)	2.28(0.131)	0.71(0.46~1.11)
Additive	CC	31(17.4)	27(13.8)	-	1
	CT	99(55.6)	102(52.0)	-	0.85(0.47~1.52)
	TT	48(27.0)	67(34.2)	2.60(0.273)	0.62(0.33~1.18)
Multiple	C	161(45.2)	156(39.8)	-	1
	T	195(54.8)	236(60.2)	2.25(0.133)	0.80(0.60~1.07)

### Linkage Disequilibrium (LD) and Haplotype Analysis

LD analysis showed that rs8073069 and rs9904341 were in strong LD, with the *r^2 ^*= 0.288, *D' *= 0.916, *D' *95%*CI *(0.830~0.960); rs8073069 and rs1042489 were also in strong LD, with the *r^2 ^*= 0.425, *D' *= 0.945, *D' *95%*CI *(0.880~0.980); rs9904341 and rs1042489 were in weak LD, with the *r^2 ^*= 0.026, *D' *= 0.189, *D' *95%*CI *(0.070~0.290). These results stated clearly the three identified *survivin *gene polymorphisms were in LD.

With the help of multivariate logistic regression, factors like drinking, HBsAg+, the medical history of viral hepatitis type B and different haplotypes constructed by rs8073069-rs9904341-rs1042489 were included into models. As shown in Table 6 the recessive genetic model was the optimal one, according to the lowest vale of Akaike's Information Criteria (*AIC*). With no G-C-T haplotype as reference, it was found that the haplotype of G-C-T from these loci was associated with a lower risk for HCC under the recessive model (*OR = *0.46, 95%*CI*: 0.24~0.90, *P *= 0.023), whereas none of other significant haplotypes were found in this model. It also indicated that HBsAg+ and the medical history of viral hepatitis type B were risk factors for HCC, with the *OR *being 27.03 (*P *< 0.001) and 55.16 (*P *< 0.001) respectively. However, no statistically significant haplotype-environment interaction was detected under different genetic models, as shown in Table 7.

**Table 6:** Analysis of haplotypes and envioronments under different genetic models

Factor	Dominant model		Recessive model		Additive model	
	*β*(*P*)	*OR*(95%*CI*)	*β*(*P*)	*OR*(95%*CI*)	*β*(*P*)	*OR*(95%*CI*)
Drinking	0.00(0.999)	1.00(0.52~1.91)	0.00(0.999)	1.00(0.52~1.91)	0.00(0.999)	1.00(0.52~1.91)
HBsAg+	3.30(< 0.001)	27.03(13.30~54.92)	3.30(< 0.001)	27.03(13.30~54.92)	3.30(< 0.001)	27.03(13.30~54.92)
H^a^	4.01(< 0.001)	55.16(7.10~428.57)	4.01(< 0.001)	55.16(7.10~428.57)	4.01(< 0.001)	55.16(7.10~428.57)
C-G-C^b^	0.12(0.624)	1.13(0.70~1.81)	0.24(0.486)	1.27(0.65~2.45)	-0.12(0.843)	0.89(0.29~2.78)
G-C-C^b^	0.56(0.031)	1.75(1.05~2.92)	-0.07(0.894)	0.93(0.32~2.69)	0.18(0.762)	1.20(0.37~3.90)
G-C-T^b^	0.07(0.775)	1.07(0.67~1.71)	-0.77(0.023)	0.46(0.24~0.90)	-0.32(0.574)	0.73(0.24~2.20)
G-G-T^b^	0.27(0.256)	1.31(0.82~2.11)	-0.17(0.652)	0.84(0.40~1.77)	-0.08(0.885)	0.92(0.30~2.86)
*AIC*	1987.66		1986.23		1988.08	

^a ^The medical history of viral hepatitis type B; ^b ^The structure of C-G-C, G-C-C, G-C-T, G-G-T haplotypes was rs8073069-rs9904341-rs1042489

**Table 7:** Analysis of haplotype-envioronment interactions under different genetic models

Factor	Dominant model		Recessive model		Additive model	
	*β*(*P*)	*OR*(95%*CI*)	*β*(*P*)	*OR*(95%*CI*)	*β*(*P*)	*OR*(95%*CI*)
HBsAg+	3.62(< 0.001)	37.41(16.36~85.53)	3.31(< 0.001)	27.33(13.46~55.48)	3.51(< 0.001)	33.41(15.32~72.84)
H^a^	3.90(< 0.001)	49.45(6.12~399.87)	3.96(< 0.001)	52.53(6.74~409.28)	3.87(< 0.001)	47.87(5.98~382.93)
G-C-T^b^	0.25(0.479)	1.06(0.65~2.53)	-0.93(0.149)	0.39(0.11~1.40)	-0.12(0.646)	0.89(0.54~1.46)
G-C-T^b^*HBsAg+	-0.59(0.122)	0.56(0.26~1.17)	-0.22(0.765)	0.80 (0.19~3.38)	-0.35(0.197)	0.70(0.41~1.20)
G-C-T^b^* H^a^	0.18(0.596)	1.19(0.62~2.30)	0.67(0.304)	0.51(0.54~7.06)	0.21(0.414)	1.23(0.75~1.64)
*AIC*	1986.19		1981.85		1983.92	

^a ^The medical history of viral hepatitis type B; ^b^The structure of G-C-T haplotypes was rs8073069-rs9904341-rs1042489; * The interaction between haplotype and environment.

## Discussion

*Suvivin *gene, located in 17q25, containing three exons and four introns, can encode an apoptosis inhibitor consisting of 142 amino acids. Nowadays, several SNPs have been identified in this gene [24]. The present case-control study explored the association of *survivin *gene polymorphisms with the risk of HCC in Chinese han population for the first time. Our results suggested that none of rs8073069, rs9904341 or rs1042489 polymorphisms in *survivin *gene correlated with the susceptibility to HCC. The polymorphism of rs1042489 is in the 3' untranslated region of *survivin *gene, studies about this polymorphism haven't been reported yet. Our result showed that rs1042489 C/T polymorphism didn't correlate with the risk of HCC, indicating this polymorphism probably had nothing to do with the stability of survivin mRNA or its translational efficiency [30]. However, additional studies are required to clarify it.

The locus of rs8073069 (-625 G/C) is located in the promoter region of *survivin *gene. There were two studies on the relationship between this polymorphism and cancers, whereas no similar studies have been conducted on HCC. Yang et al. [25] launched a case-control study in Chinese population identifying that rs8073069-C allele was a risk factor for esophageal squamous cell carcinoma (ESCC), with the *OR *of CC genotype being 2.404 in contrast to GG genotype. Yang also described different survivin expression levels between subgroups with different rs8073069 G/C variants in ESCC patients. His study suggested that rs8073069 G/C polymorphism was associated with the susceptibility to ESCC, perhaps by influencing survivin expression. However, Jang et al. [26] discovered that rs8073069 G/C polymorphism was not linked to the risk of lung cancer in Korea population. Our study indicated that rs8073069 G/C polymorphism didn't correlate with the HCC in Chinese han population, this was consistent with Jang's results, but inconsistent with Yang's study. One suggestive explanation was that genetic susceptibility may be different to diverse cancers, other molecular and cellular mechanisms were probably involved in survivin overexpression in HCC. Besides, the negative results were perhaps due to the relatively small sample size, and therefore additional studies with lager samples are needed to validate our finding.

The locus of rs9904341 (-31 C/G) is located in *survivin *gene promoter. Several studies on the association of this polymorphism with cancers have been carried out. However their results were inconsistent. Jang et al. [26] discovered that individuals with at least one rs9904341-G allele had a significantly decreased risk for lung cancer compared to those with CC genotype. Promoter assay revealed G allele had a lower promoter activity than rs9904341-C allele. His results indicated this polymorphism regulated survivin expression, and thus modulated susceptibility to lung cancer. Maria et al. [31] found the association of this polymorphism with the risk of the sporadic colorectal cancer (CRC). Moreover, homozygotes for rs9904341-CC genotype expressed 1.6-fold higher mRNA levels of survivin compared to cases with other genotypes. Nonetheless, no correlation was found of rs9904341 C/G polymorphism with some cancers including ESCC, cervical cancer and acute myeloid leukemia [25,32,33]. In addition, Bayram et al. [28] demonstrated that there was no statistical association of rs9904341 C/G polymorphism with the risk of HCC in Turkish population. These inconsistent results may be attributable to differences in the pathways of carcinogenesis among various types of human cancers. The present study documented that rs9904341 was not associated with the risk of HCC in Chinese han population, it's in line with Borbely's study, suggesting that rs9904341 didn't correlate with the risk of HCC. However, the negative result was perhaps due to the small sample size, so replication of this finding in larger samples is needed.

Haplotype analysis can obtain more information than single SNP and thus elevates the statistical power by making an assay of several SNPs within the same gene simultaneously [34]. LD analysis pointed out that these loci were in LD, therefore we made an assay of haplotypes constructed by the identified polymophisms. With no G-C-T haplotype as reference, the haplotype of G-C-T was associated with a lower risk for HCC under the recessive genetic model (*OR = *0.46, 95%*CI*: 0.24~0.90). This suggested G-C-T haplotype was perhaps a protective genetic factor for HCC in Chinese han population. Nonetheless, it requires further studies to confirm. Both HBsAg+ and the medical history of viral hepatitis type B were risk factors for HCC, whereas no statistically significant haplotype-environment interaction existed.

To date, only one similar study on the correlation of *survivin *gene with HCC has been reported. Since polymorphisms often vary among different ethnic groups, additional and lager sample size studies are required to validate the association of *survivin *gene polymorphisms with HCC in diverse ethnic populations. However, our study was considered credible, suggesting *survivin *gene polymorphisms (rs8073069, rs9904341 or rs1042489) didn't correlate with HCC, at least in Chinese han population.

## Conclusions

In conclusion, this is the first report regarding the association of the *survivin *gene polymorphisms with the risk of HCC in Chinese han population. No association between rs8073069, rs9904341 or rs1042489 polymorphisms in *survivin *gene and the risk of HCC is found in the present study, but rs8073069G-rs9904341C-rs1042489T is perhaps a protective haplotype for HCC in Chinese han population. Further studies will be needed to see whether *survivin *gene polymorphism has a role in HCC in other geographical region.

## Competing interests

The authors declare that they have no competing interests.

## Authors' contributions

The study's chief researcher WM was responsible for identifying the research question, designing the study, obtaining ethics approval and overseeing the study. WL and FJ were in charge of the study fields, collecting subjects' data. YL and JW majored in the laboratorial work, analyzing the data, writing the manuscript. All authors were responsible for drafting the manuscript, read and approved the final version.

## Pre-publication history

The pre-publication history for this paper can be accessed here:

http://www.biomedcentral.com/1471-2350/13/1/prepub
